# Prevalence and Factors Associated with Musculoskeletal Disorders among Secondary Schoolteachers in Hail, Saudi Arabia: A Cross-Sectional Survey

**DOI:** 10.3390/ijerph18126632

**Published:** 2021-06-20

**Authors:** Omar W. Althomali, Junaid Amin, Wael Alghamdi, Daria Hussain Shaik

**Affiliations:** 1 Department of Physiotherapy, College of Applied Medical Sciences, University of Ha’il, Ha’il 2440, Saudi Arabia; o.althomali@uoh.edu.sa (O.W.A.); sd.hussain@uoh.edu.sa (D.H.S.); 2 Department of Nursing, College of Applied Medical Sciences, AlBaha University, AlBaha 1988, Saudi Arabia; waelalghamdi@bu.edu.sa

**Keywords:** occupational health, pain, quality of life, sex differences, risk factors, teachers, work-related musculoskeletal disorders (WMSDs), work

## Abstract

Musculoskeletal disorders (MSDs) are one of the most common occupational health hazards and serious health concerns among teachers. About 39% to 95% of teachers suffer from musculoskeletal pain that can lead to a decline in their performance, frequent sick leaves and early retirement, and can have a negative impact on their quality of life. The aim of this study was to investigate the prevalence of and risk factors for MSDs among secondary schoolteachers in Hail, Saudi Arabia. A cross-sectional study was conducted through an electronic survey. A self-reported and validated Arabic version of the Nordic Musculoskeletal Questionnaire (NMQ) was used. Participants were recruited randomly through a two-stage sampling technique. A total of 251 respondents (57.8% males and 42.2% females) returned the questionnaire. The overall prevalence of MSDs was 87.3%. Female teachers (95.3%) suffered more than their male counterparts (81.4%). The most common site was the lower back (62.55%), followed by the shoulders (53.39%) and knees (41.04%). Most of the participants (72.7%) reported MSDs in multiple sites. Binomial logistic regression predicted that smoking is a significant risk factor for lower back disorders (*p* < 0.05). A high prevalence of MSDs can be due to a substantial lack of awareness and practice of ergonomics, which warrant the development of preventive strategies and educational programs.

## 1. Introduction

Musculoskeletal disorders (MSDs) are injuries affecting the muscles, ligaments, tendons, nerves, bones and joints [1,2]. MSDs are recognised as one of the most common occupational health hazards in the working environment negatively affecting the quality of life of working professionals [3]. Work is an essential part in the life of every individual [4,5]. Teachers play a vital role in the effective functioning of the educational system and for the improvement of the quality of learning processes [6,7]. Nonetheless, MSDs have become a health hazard caused either by the work itself or by the teaching environment [8]. Epidemiological studies predict that almost 39% to 95% of adults in the teaching profession are affected by musculoskeletal pain during their professional careers [6,9,10].

The work of schoolteachers often involves a head-down posture such as reading, using a computer and grading schoolwork [4]. The teaching profession is not only limited to teaching students but also involves getting ready for lessons, assessing students’ homework and taking part in school activities. Sometimes, teaching is carried out in an unsuitable environment, during which teachers apply their physical, cognitive and emotional capacities to reach their teaching goals [6]. A bad posture of the body while teaching and communicating with students is a potential risk factor for MSDs in teachers that has a negative impact on quality of work [11].

Initial studies focused on identifying the cause of musculoskeletal complaints with psychological changes and physical activities at work among schoolteachers [12,13]. These studies revealed that teachers with musculoskeletal complaints are more prone to psychological stress. The studies conducted in Saudi Arabia and around the globe also showed a high prevalence of MSDs and the involvement of multiple anatomical sites [7,11,14,15,16]. MSDs are core factors that have a negative impact on work performance; hence, they may lead to frequent sick leaves and early retirement for teachers, which will hamper the quality of the educational system [12]. Therefore, MSDs tend to lead to physical stress and mental health problems, causing a negative impact on the quality of life of teachers [11].

Although a lot of research studies around the world have revealed that teachers are at a high risk of MSDs, only a few studies have addressed MSDs among schoolteachers in Saudi Arabia [14,15,16]. Moreover, these studies involved either male or female teachers. To date, not a single study on MSDs among schoolteachers from Saudi Arabia included both male and female subjects. There was an apparent need to address the MSDs in both male and female secondary schoolteachers. Moreover, the previous studies mentioned different working conditions for men and women. These studies also reported that women are less qualified than men, with lower salaries, higher demands and less control over their work [14]. The Sex and Gender Equity in Research (SAGER) guidelines also support our inclusion of both genders. In this study, we aimed to investigate the prevalence and factors associated with MSDs in secondary schoolteachers in Hail, Saudi Arabia.

## 2. Materials and Methods

### 2.1. Study Design and Participants

A quantitative, observational study with a cross-sectional design was conducted among secondary schoolteachers of Hail, Saudi Arabia. Teachers of both genders with at least one year of work experience in government secondary schools and currently on the job were included in this study. Those with a physical disability, malignant tumours and inflammatory joint diseases (rheumatoid arthritis, gout and ankylosing spondylitis), those who were pregnant or had previous surgery in the limbs and spine and those who work in private schools or have retired were excluded.

### 2.2. Sampling

**Figure 1:**
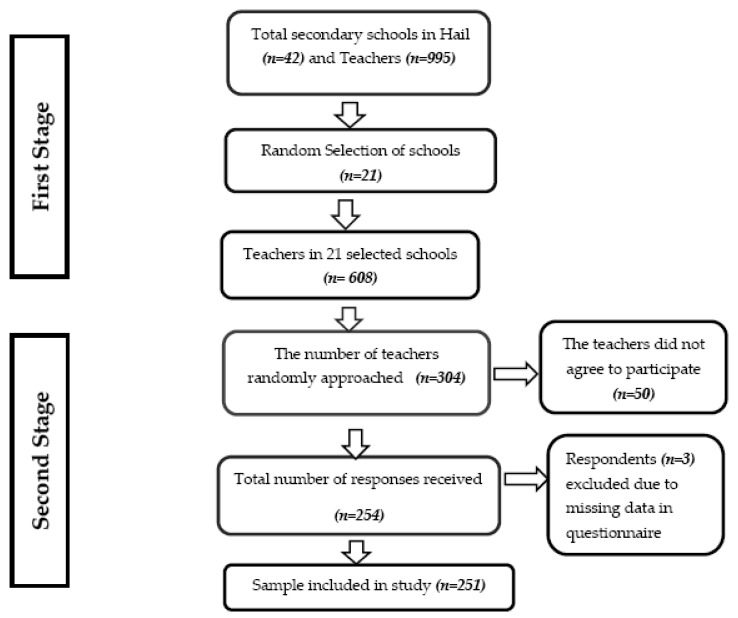
Flow diagram of sample selection.

The sample size was calculated with a confidence interval of 95% at a response rate of 70% and a marginal error of 5%. The required sample size was 244 that was calculated using an online Raosoft sample size calculator (Raosoft, Inc., Seattle, WA, USA). The recruitment of the participants involved a two-stage sampling process, namely, a random sampling of schools followed by random selection of secondary schoolteachers from those schools. According to the Ministry of Education, the number of secondary schools in Hail was 42 in 2017, and the number of secondary schoolteachers was 995. During the first sampling stage, a list of 42 schools was prepared and every second school was randomly selected. The total number of teachers in 21 selected schools was 608. Of those 608 teachers, every second teacher was randomly selected, giving a sample size of 304, and 254 returned the questionnaire, giving a response rate of 83.55%. Three respondents were excluded because of missing data. Finally, 251 respondents were included in this study (Figure 1). 

### 2.3. Instrument

The data on demographics, lifestyle and work characteristics were collected prior to administering the main study questionnaire, which included gender, age, marital status, smoking habits, years of experience and daily hours of sleep and work. These variables were selected from the reviewed literature about the possible risk factors for MSDs. Gender and age have been identified as risk factors for MSDs in a systematic review among teachers [6]. Interestingly, several factors have been positively associated for MSDs such as long working hours [6,14], length of employment duration [4,6], smoking [17], marital status [18] and sleeping hours [19].

The responses about MSDs among schoolteachers using a self-reported and validated Arabic version of the Nordic Musculoskeletal Questionnaire (NMQ) were collected [14]. NMQ is valid, reliable and responsive [20,21]. Both English and Arabic versions of the NMQ have been used in several studies for the analysis of musculoskeletal symptoms in an occupational health context [18]. This questionnaire addresses three main parts. The first part is about any trouble (such as pain, ache, discomfort and numbness) felt by the respondent in the last 12 months. The second part asks the same question but for the last seven days. The final part is about the disability caused by the trouble in the last 12 months. In each part, the data collected were about different anatomical areas: neck, upper back, lower back, shoulders, elbows, wrists/hands, hips/thighs/buttocks, knees and ankle/feet. The responses were recorded in the form of binary options (“yes” and “no”).

### 2.4. Data Collection

The data were collected electronically by using an online Google form after taking the participants’ informed consent. The link of the survey was shared with the potential participants electronically on WhatsApp and by email. Two reminders were sent after a three-day interval to increase the response rate.

### 2.5. Ethical Considerations

Ethical approval was obtained from the research ethics committee of the University of Hail (H-2020-007). Informed consent was included in the online form before the main survey questions began. The answers to the survey questions were anonymous, and the collected data were kept confidential.

### 2.6. Statistical Analyses

The collected data were downloaded from the online Google form as a Microsoft Excel sheet (version 16.33), then exported to SPSS version 25 (SPSS, Inc., Chicago, IL, USA). The data relating to the trouble felt in the last seven days were analysed as it could provide more reliable information by minimising memory recall bias. Categorical data were presented in the form of frequencies and percentages. Microsoft Excel was used to construct a bar chart. The data were also presented with gender as a factor. The association between gender and different anatomical sites was assessed by using a chi square test. To determine if the anatomical sites differed with respect to troubles in the last seven days, Cochran’s Q test was used. Binomial logistic regression was used to predict demographics and lifestyle characteristics related to the three most common sites of trouble during the last seven days. Each variable was faded to the regression model alone. The statistical significance level was considered below 0.05.

## 3. Results

A total of 251 participants were included in this study, with slightly more males (57.8%) than females (42.2%). Almost half of the participants were between 36 and 45 years of age. Most of the respondents were married (85.7%), 53% had 7–8 of sleeping hours per day and only 21.9% were smokers. Moreover, 36.3% of the responders had more than 20 years of teaching experience, and 215 (85.6%) worked between 5 to 8 h daily. The basic characteristics of the study participants are shown in Table 1.

The overall prevalence of MSDs was 87.3% (219 of 251 teachers). The prevalence of MSDs among males was 81.4% (118 of 145 teachers) and that among females was 95.3% (101 of 106 teachers) (Table 2). The most common sites of MSDs were the lower back (157; 62.55%), followed by the shoulders (134; 53.4%), knees (103; 41.04%), hips/thighs/buttocks (93; 37.05%) and neck (91; 36.25%). The least common sites reported were the elbow (41; 16.3%), followed by the wrists/hands (76; 30.3%) and ankles/feet (79; 37.05%) with differences statistically significant at the different sites χ2(8) = 143,209.508951, *p* < 0.0005.

**Figure 2:**
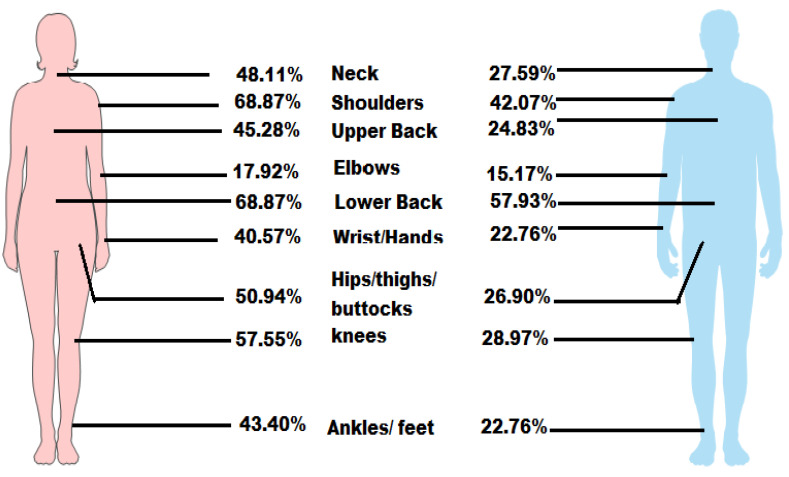
Gender-based distribution of musculoskeletal disorders in teachers.

The most common site of MSDs among males was the lower back (84; 57.9%), followed by the shoulders (61; 42.1%), whereas the least common site was the elbows (22; 15.17%). The most common sites of MSDs among females were the lower back (73; 68.9%) and shoulders (73; 68.9%), whereas the least common site was the elbow (19; 17.9%). The gender-based trend of MSDs is shown in Table 3 and Figure 2.

The association between gender and anatomical sites of MSDs was examined. MSDs symptoms of all body sites were worse in females than in males. Moreover, MSDs of some anatomical sites were found to be significant between gender, such as neck [χ2(1, *n* = 251) = 13.232 *p* = *0*.000], shoulders [χ2(1, *n* = 251) = 17.672 *p* = *0*.000], wrist/hands [χ2 (1, *n* = 251) = 9.198 *p* = *0*.002], upper back [χ2(1, *n* = 251) = 11.507 *p* = *0*.001], hips/thighs/buttocks [χ2(1, *n* = 251) = 15.182 *p* = *0*.000], knees [χ2(1, *n* = 251) = 20.674 *p* = *0*.000] and ankles/feet [χ2(1, *n* = 251) = 12.09 *p* = *0*.001] (Table 3).

Binomial logistic regression was performed to investigate the effects of age, gender, smoking, sleeping hours per day, experience, working hours per day and lower back, shoulder and knee disorders. The model explained 8.2% (Nagelkerke R2) of variations in lower back pain and correctly classified 66.5% of the cases. Of the seven variables included, only smoking was statistically significant “Odds ratio (OR): 2.64 (Confidence Intervals 95% (95% CI): 1.36–5.13; *p* = 0.004)”. Smokers had a 2.64-fold higher odds ratio than nonsmokers (Table 4). In the shoulder region, the model explained 11.6% (Nagelkerke R2) of variations in shoulder pain and correctly classified 65.3% of the cases. Gender was statistically significant “OR: 2.697 (95% CI: 1.524–4.776; *p* value = 0.001)”. Females had a 2.697-fold higher odds ratio than males (Table 5). In the knee region, the model explained 16.1% (Nagelkerke R2) of the variations in knee pain and correctly classified 67.7% of the cases. The association of knee pain with gender and sleeping hours was statistically significant: gender “OR: 3.649 (95% CI: 2.009–6.630; *p* = 0.000)” and sleeping hours “OR: 0.689 (95% CI: 0.477–0.996; *p* = 0.048)”. Females had a 3.649-fold higher odds ratio than males (Table 6).

## 4. Discussion

In this study, we aimed to determine the prevalence of, and factors associated with MSDs among secondary schoolteachers in Hail, Saudi Arabia. The teaching profession is included among the professions that is more prone to suffer from MSDs [22,23], and only a few studies focused on MSDs in Saudi schoolteachers [14,15,16]. The findings of this study showed that the prevalence of MSDs among Saudi schoolteachers is high. More than two-thirds of the participants in this study had complained of MSDs in multiple parts of the body. The lower back and shoulders were the most prevalent sites, followed by knee joints. Prevalence of MSDs was more in female teachers than their male counterparts. MSDs represent one of the most common occupational health hazards in the working environment. Musculoskeletal pain is the main cause of absenteeism from work, decreased quality of life and early retirement of schoolteachers, limiting physical and professional functions and, finally, causing a huge economic loss for the state.

The prevalence of MSDs (87.3%) among secondary schoolteachers was higher than that in previous studies. A study conducted in Abha during 2020 explored that the rate of MSDs reported among teachers was 62.5% [16], whereas other studies conducted in Turkey and China showed similar values of 60.3% and 66.7%, respectively [6,24]. The MSDs problems found in our study was also higher than in other similar studies conducted in Turkey (28.0%) [25] and China (31.8%) [4], but close to those in a Saudi Arabian study conducted among female secondary school teachers in Dammam (79.17%) [14] and in Turkey (77%) [26] and China (77%) [27]. Previous studies from different countries have also highlighted these findings, which reported a significant occurrence of MSDs among schoolteachers. The prevalence found in this study was also in line with a systematic review on MSDs among teachers that included studies from different parts of the world, which showed prevalence of MSDs between 39% and 95% [6]. Likewise, the finding was inconsistent with studies conducted among dentists [22], physiotherapists [23] and nurses [28], which reported a higher prevalence of MSDs like schoolteachers in this study. The prevalence among females was 75.5%, which is relatively less than that among female teachers in Dammam (79.17%) [14]. The prevalence among males was 81.4%, which is much higher than that among male teachers in Abha (62.5%) [16]. This high rate of reported MSDs (87.3%) might be due to schoolteachers are bombarded with various activities at the workplace, and from long hours of standing and awkward posture. Moreover, secondary schoolteachers have more pressure regarding the performance of graduating students and are involved with more examinations. Professional discomforts may be produced from constantly spending time in inappropriate body mechanics at the workplace, such as leaning forward, twisting of back, long-term period of standing, computer use each day and bad body posture during teaching [11].

The current study showed that female experience more MSDs than male in most of the anatomical regions. MSDs were also found positively associated with females for shoulder and knee. Moreover, being female increases the likelihood of developing knee and shoulder disorders. These findings are in line with a study conducted in China [7]. Clinical studies showed some sex-related clinical signs of musculoskeletal disorders [29]. Previous studies showed that females experience higher pain intensity, more disability and greater pain interference with function compared to males [30]. Additionally, females report worse anxiety, depression and self-efficacy [31]. Several biochemical and biological differences have been identified related to the previously mentioned differences. Females have more mechanically sensitive C and Aδ afferents which response to mechanical distortion, in their muscles than males [30]. In addition, females appear to have higher cytokine production response (stronger inflammatory response) to tissue damage than males [29].

The present study also showed that the most prevalent site was the lower back (62.55%). These results are similar to those of different Saudi Arabian studies from Abha (59.2%) [16] and Dammam (63.8%) [14] and a national-based survey (66.9%) [15]. It was also in line with studies reported from other countries. The prevalence of lower back pain was much higher in this study than in those conducted in China (45.6%) [27], Brazil (41.1%) [32], Turkey (38%) [26] and Malaysia (40.4%) [32]. In contrast, a Japanese study showed less prevalence of back pain and reported that only 20.6% of schoolteachers suffered from back pain [6]. Moreover, the lower back has also been reported as the most commonly affected area in other professionals, including dentists (79%) [22,33], physiotherapists (68.8%) [23] and nurses [28]. In our study, females suffered more with lower back problems (69.9%) as compared to males (57.9%) and Chinese female reported the same findings [7]. The cause of back pain is usually overloading and overuse of the spine. Teachers are prone to an awkward posture and twisting of the back while using computers, grading assignments, reading frequently and standing for long hours while delivering lectures. These activities might have led to back pain and other physical problems while performing a variety of job functions. In this regard, a systematic review by Lis et al. (2007) showed that prolonged sitting with an awkward posture and twisting of the back increase the risk of lower back pain. Evidence suggests that there is a relationship between back pain and advancing age, and because of this, middle-aged teachers are more susceptible to back pain that interferes with their social, professional and physical function [34].

The association of back pain with potential risk factors was determined, and only smoking was significantly associated with the lower back disorder. Previous studies have also shown a significant association between smoking and lower back pain, which support our findings and attributed this to biological factors associated with smoking [35].

Several factors can be highlighted as possible mechanisms of association between smoking and MSDs. Recent systematic review showed that smoking is associated with lower bone mineral density, periodontitis, increased fracture risk, tooth implant failure and alveolar bone loss. The review reported that at the joint level, smoking has been found to increase joint disease activity, poor therapeutic effect, and poor functional outcome. Moreover, smoking was also associated with muscle, cartilage, tendons and ligaments adverse effects [36]. The pathogenesis of such issue is complex, due to direct toxic materials (nicotine) on the osteoclasts/osteoblasts activity and indirect effect on adrenocortical hormones and sex, vessels, calcium absorption and oxygen supply [37]. Other factors were not associated with the lower back disorder. However, ergonomics, bad posture and other factors were not investigated, and these may be more important than counting the total working or sleeping hours.

Our study also revealed that shoulder pain (53.39%) was the second most common problem among teachers. Notably, this prevalence was consistent with the findings of different Saudi Arabian studies conducted in Abha (47.9%) [16] and Dammam (45.4%) [14] but contrasting with a Saudi national survey that reported a shoulder pain prevalence of only 20.6% [15]. There was a significant association between shoulder disorders and gender. Females were suffering more with shoulder problems as compared to males. The high reported problems of shoulder disorders (69.9%) among females are comparable with Chinese female schoolteachers that was 73.4% [7]. The professionals who suffered from shoulder pain had a convincing reason because they use a computer for a long time each day. A static work posture with elevated arms while using a whiteboard and the use of a computer with a slouched posture contribute to humeral compression against the coracoacromial arch, resulting in impaired circulation to shoulder tendons. Likewise, repetitive movements of upper extremities also contribute to overuse injuries of the shoulder tendons and impairment of upper extremity functions [38,39].

The prevalence of knee pain was 41.04%, which was reported as the third most common disorder in our study. A significant association of knee pain with gender and daily hours of sleep was also established. These findings are almost also in line with those of similar studies conducted among schoolteachers in Abha [16], and Dammam [14] with knee disorder prevalences of 43.3% and 40.0%, respectively.

Nevertheless, this study contributes useful information to the body of knowledge on MSDs problems among schoolteachers with few limitations. This study used a cross-sectional design that has limitations on reporting casual association and the possibility of reverse causation. The cross sectional study only provides weak evidence between the exposure and outcome as it is too complicated to separate the cause and effect. It is difficult to determine whether disease followed the preceded exposure or not. Moreover, owing to the lack of logistical support; we were able to plan only a survey-based study, which is an inexpensive and easy method of data collection. A self-reported survey can have some reporting bias as well.

## 5. Conclusions

We found a high prevalence of MSDs among teachers, which represents a substantial lack of awareness about ergonomics and warrants the development of preventive strategies and educational programs to reduce the burden of MSDs among susceptible professions in general and teachers in particular. Our findings are helpful for further studies that explore gender-specific occupational, psychosocial and personal risk factors. Furthermore, studies that focus on the economic impact of MSDs and effective strategies to prevent MSDs among teachers may be a good contribution to the scientific literature. Likewise, different interventional models should be introduced as part of preventive strategies to reduce the prevalence of MSDs at the most commonly affected sites.

## Figures and Tables

**Table 1:** Demographics, lifestyle and work characteristics of the participants (*n* = 251).

Characteristics	Sub-Division	*n* (%)
Gender	Male	145 (57.8)
	Female	106 (42.2)
Age (years)	26–30	30 (12.0)
	31–35	23 (9.2)
	36–40	59 (23.5)
	41–45	61 (24.3)
	46–50	50 (19.9)
	>50	28 (11.2)
Working hours per day	2–4	15 (6.0)
	5–6	95 (37.8)
	7–8	120 (47.8)
	9–10	14 (5.6)
	>10	7 (2.8)
Marital status	Never married	28 (11.2)
	Married	215 (85.7)
	Widowed	6 (2.4)
	Divorced	2 (0.8)
Smoking habits	Smokers	55 (21.9)
	Non-smokers	196 (78.1)
Sleeping hours per day	2–4	10 (4.0)
	5–6	79 (31.4)
	7–8	133 (53.0)
	9–10	26 (10.4)
	11–12	1 (0.4)
	>12	2 (0.8)
Job experience	1–5	22 (8.8)
	6–10	43 (17.1)
	11–15	51 (20.3)
	16–20	44 (17.5)

**Table 2:** Prevalence of musculoskeletal disorders (*n* = 251).

Variables	*n* (%)
Musculoskeletal disorders (MSDs)	
Yes	219 (87.3)
No	32 (12.7)
Gender	
Male	118 (81.4)
Female	101 (95.3)
Number of anatomical Sites	
No	32 (12.7)
Single	37 (14.6)
Multiple	182 (72.7)

**Table 3:** Anatomical distribution of musculoskeletal symptoms and association of anatomical sites with gender.

Anatomical Site	Total *n* (%)	Male *n* (%)	Female *n* (%)	*p* Value
Neck	91 (36.25)	40 (27.59)	51 (48.1)	0.001 *
Shoulders	134 (53.4)	61 (42.1)	73(68.9)	0.00 *
Elbows	41 (16.3)	22 (15.2)	19 (17.9)	0.56
Wrist/hands	76 (30.3)	33 (22.8)	43 (40.6)	0.002 *
Upper back	84 (33.5)	36 (24.8)	48 (45.3)	0.001 *
Lower back	157 (62.55)	84 (57.9)	73 (68.9)	0.077
Hips/thighs/buttocks	93(37.05)	39 (26.9)	54 (50.9)	0.00 *
Knees	103(41.04)	42 (29.0)	61 (57.55)	0.00 *
Ankles/feet	79(31.5)	33 (22.8)	46 (43.4)	0.001 *

* *p* value obtained from chi-square test.

**Table 4:** Binomial regression results for lower back disorder with different study characteristics.

Variables in the Equation	B	S.E.	Wald	df	Sig.	Exp(B)	95% C.I. for EXP(B)	
							Lower	Upper
Gender (female)	0.190	0.300	0.403	1	0.525	1.210	0.672	2.175
Smoking (yes)	0.970	0.340	8.151	1	0.004	2.638	1.355	5.134
Age	0.121	0.147	0.675	1	0.411	1.128	0.846	1.505
Years of experience	−0.021	0.158	0.018	1	0.895	0.979	0.719	1.334
Working hours per day	−0.146	0.170	0.738	1	0.390	0.864	0.619	1.206
Sleeping hours per day	−0.048	0.176	0.075	1	0.785	0.953	0.675	1.345
Marital status	0.365	0.335	1.185	1	0.276	1.440	0.747	2.778
Constant	−0.684	0.827	0.684	1	0.408	0.505		

**Table 5:** Binomial regression results for shoulder disorder with different study characteristics.

Variables in the Equation	B	S.E.	Wald	df	Sig.	Exp(B)	95% C.I. for EXP(B)	
							Lower	Upper
Gender (female)	0.992	0.291	11.593	1	0.001	2.697	1.524	4.776
Smoking (yes)	0.389	0.342	1.295	1	0.255	1.475	0.755	2.881
Age	−0.132	0.144	0.848	1	0.357	0.876	0.661	1.161
Years of experience	0.076	0.156	0.237	1	0.626	1.079	0.795	1.463
Working hours per day	0.180	0.169	1.137	1	0.286	1.197	0.860	1.666
Sleeping hours per day	−0.134	0.177	0.572	1	0.450	0.875	0.619	1.237
Marital status	−0.208	0.328	0.402	1	0.526	0.812	0.427	1.546
Constant	−1.260	0.813	2.400	1	0.121	0.284		

**Table 6:** Binomial regression results for knee disorder with different study characteristics.

Variables in the Equation	B	S.E.	Wald	df	Sig.	Exp(B)	95% C.I. for EXP(B)	
							Lower	Upper
Gender (female)	1.295	0.305	18.058	1	0.000	3.649	2.009	6.630
Smoking (yes)	−0.187	0.363	0.267	1	0.605	0.829	0.407	1.687
Age	0.069	0.150	0.213	1	0.645	1.072	0.798	1.439
Years of experience	0.196	0.160	1.499	1	0.221	1.217	0.889	1.666
Working hours per day	0.048	0.173	0.078	1	0.779	1.050	0.748	1.474
Sleeping hours per day	−0.372	0.188	3.917	1	0.048	0.689	0.477	0.996
Marital status	0.080	0.359	0.049	1	0.824	1.083	0.536	2.190
Constant	−2.237	0.871	6.598	1	0.010	0.107		

## Data Availability

The data presented in this study are available within the article.
